# The use of extended-release tacrolimus twice a day might be beneficial for selected kidney transplant recipients: a case report

**DOI:** 10.3389/fmed.2024.1336035

**Published:** 2024-06-26

**Authors:** Louise Füessl, Lena Kreuzer, Kajetan Nierychlewski, Tobias Seibt, Manfred Johannes Stangl, Dionysios Koliogiannis, Bruno Meiser, Markus Schwarz, Michael Fischereder, Stephan Kemmner

**Affiliations:** ^1^Transplant Center, University Hospital Munich, Ludwig-Maximilians-University (LMU), Munich, Germany; ^2^Division of Nephrology, Department of Medicine IV, University Hospital Munich, Ludwig-Maximilians-University (LMU), Munich, Germany; ^3^Department of Nephrology and Rheumatology, Augustinum Klinik München, Munich, Germany; ^4^Institute of Laboratory Medicine, University Hospital Munich, Ludwig-Maximilians-University (LMU), Munich, Germany; ^5^Department of General, Visceral and Transplantation Surgery, University Hospital Munich, Ludwig-Maximilians-University (LMU), Munich, Germany

**Keywords:** tacrolimus, kidney transplantation, calcineurin inhibitor toxicity, extended-release tacrolimus, prolonged-release tacrolimus

## Abstract

The calcineurin inhibitor tacrolimus, which is available as an immediate- or extended-release formulation, is the standard-of-care immunosuppression after kidney transplantation with low rejection rates, especially in the first year after transplantation. However, its highly variable metabolism rate, narrow therapeutic window, and nephrotoxic side effects require close drug monitoring and individual dosing. Here, we describe first the application of extended-release tacrolimus (ER-Tac) twice daily with beneficial effects in a kidney transplant recipient under extensive therapeutic drug monitoring. A 47-year-old female kidney transplant recipient, who was identified as a fast metabolizer for tacrolimus, presented with declining allograft function and low tacrolimus through levels over time and 8 years after a second kidney transplantation despite the administration of high doses of ER-Tac once daily. Therefore, the area under the concentration–time curve (AUC) showed exceedingly high blood levels of ER-Tac. The latest biopsy of the kidney transplant showed arteriolar hyalinosis with pole vessel stenosis as a sign of chronic transplant vasculopathy and transplant glomerulopathy as a sign of chronic humoral rejection. After the exclusion of other options for immunosuppressive therapy due to the patient’s high immunological risk, the patient was switched from ER-Tac once daily to ER-Tac twice daily. After switching to ER-Tac twice daily, the AUC for oral tacrolimus decreased and the transplant function improved despite higher tacrolimus trough levels and a lower total dose administered. This case highlights the importance of careful therapeutic drug monitoring with the performance of an AUC in the follow-up management of kidney transplant recipients.

## 1 Introduction

The calcineurin inhibitor (CNI) tacrolimus either as prolonged-, extended-, or immediate-release administration is part of the standard-of-care immunosuppressive therapy after kidney transplantation (1). Therefore, the acute rejection rates in tacrolimus-based immunosuppressive therapy, especially in the first year after kidney transplantation, are lower in contrast to other immunosuppressive regimes (2). However, a typical side effect of CNIs like tacrolimus is nephrotoxicity due to vasoconstriction. Histological lesions such as arteriolar hyalinosis can be associated with chronic CNI nephrotoxicity (3). Furthermore, the highly variable metabolism rate of tacrolimus and its narrow therapeutic window require close drug monitoring and individual dosing (4). Fast tacrolimus metabolism is associated with reduced kidney transplant function and survival, the tacrolimus metabolism rate being defined as the drug trough concentration (C) normalized by the corresponding daily tacrolimus dose (D) (5, 6). Therefore, a C/D ratio of <1.05 ng/mL*1/mg indicates fast tacrolimus metabolism (6).

Tacrolimus is available as an immediate-release formulation, which must be given twice a day, or as an extended- or prolonged-release formulation, which should normally be given once daily (1). The application of extended-release tacrolimus (ER-Tac) twice a day with a possible positive effect, especially in “fast tacrolimus metabolism,” has not been described so far (7).

## 2 Case description

We report the case of a 47-year-old female kidney transplant recipient who presented with declining allograft function 8 years after a second kidney transplantation and the resulting unusual application of extended-release tacrolimus twice daily (BID).

Kidney transplantation had previously been performed after HLA-incompatible living donation with a donor-specific antibody (DSA), specifically anti-HLA DQ7, HLA-mismatch of 1-1-2, and 82% panel-reactive antibodies. The initial immunosuppression included rituximab, plasmapheresis, and anti-thymocyte globulin (ATG) due to high immunological risk, as well as immediate-release tacrolimus (IR-Tac), mycophenolate mofetil, and prednisolone in the follow-up. Over time, the pre-known DSAs were detectable with high signal intensity (MFI approximately 20.000) for years despite the administration of tacrolimus, high-dose antimetabolite [mycophenolate mofetil (CellCept) 1 g twice daily], and persistent steroid administration (prednisolone 5 mg once daily) in this patient.

The dosage of mycophenolate mofetil and prednisolone was continued at this dose due to the immunological risk and was not changed over time.

**Figure 1:**
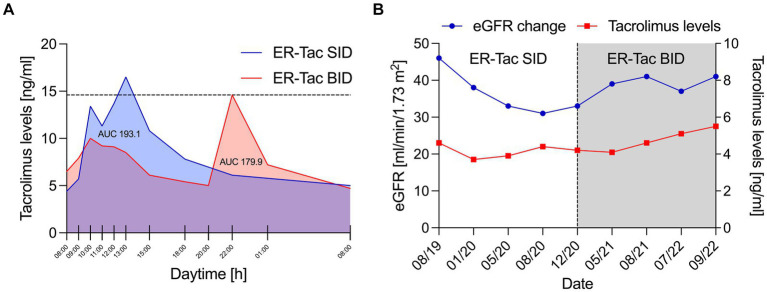
**(A)** Serum levels of tacrolimus [ng/ml] over 24 h. Blue line: Serum levels of extended-release tacrolimus (ER-Tac) once daily (SID, 12 mg); red line: serum levels of extended-release tacrolimus (ER-Tac) twice daily (BID, 2 × 5 mg). The AUC (area under the concentration–time curve) was calculated using PRISM by GraphPad. The time points used to calculate the AUC are marked on the *x*-axis. **(B)** Estimated glomerular filtration rate (eGFR) [ml/min/1.73m^2^] according to the Chronic Kidney Disease Epidemiology Collaboration (CKD-EPI) (blue line) and extended-release tacrolimus (ER-Tac) trough levels over time (red line). The black dashed line marks the switch from ER-Tac once daily (SID) to ER-Tac twice daily (BID).

To reduce peak levels of calcineurin inhibitors, IR-Tac twice a day was switched to extended-release tacrolimus (ER-Tac) once daily during long-term follow-up (8). After that, ER-Tac trough levels were in the lower range despite the administration of high doses (12 mg daily), a normal BMI (20 kg/m^2^) with a body weight of 60 kg, and correct administration, which was critically assessed by anamnesis regarding medication intake and eating behavior as well as review of concomitant medication. A C/D ratio of <1.05 ng/mL*1/mg revealed fast tacrolimus metabolism in this patient. According to the relatively high dose, the area under the concentration–time curve (AUC) showed exceedingly high blood levels of ER-Tac (Figure 1A).

There was a history of a previous graft biopsy 7 years after transplantation in this patient which revealed a mild cellular reaction (BANFF borderline), discrete signs of glomerulitis (without C4d positivity in immunohistochemistry), and transplant glomerulopathy (verified by electron microscopy) according to the BANFF lesion scores (t1, i2, v0, g1, ptc0, ci1, ct1, cv2, cg1b, mm1, ah1, aah0, ti2, and i-IFTA1). Based on these findings, the patient was treated with high-dose steroids and immunoglobulins.

Over time, the patient presented with declining eGFR and increased proteinuria. An indication biopsy performed again showed arteriolar hyalinosis with pole vessel stenosis as a sign of chronic transplant vasculopathy, which is likely aggravated by calcineurin inhibitor toxicity, as well as further evidence of transplant glomerulopathy, which is likely associated with chronic (non-active) antibody-mediated rejection (ABMR) in the absence of histological signs of acute rejection (9).

Switching to a selective co-stimulation blockade of T-cell activation with belatacept or to a mammalian target of rapamycin (mTOR) inhibitor-based regimen was not considered a suitable option because of the patient’s high immunological risk (2, 10).

To reduce the progress of worsening allograft function due to calcineurin inhibitor toxicity and to achieve an optimized therapeutic drug level, the administration of ER-Tac twice daily in adjusted dosage was prescribed after discussion of all possible options of immunosuppressive therapy and after informing the patient in detail about the unusual use. After switching to ER-Tac twice daily (BID), the AUC for oral tacrolimus (Figure 1A) and the transplant function fortunately improved despite higher tacrolimus trough levels and a lower total dose administered (Figure 1B). The patient tolerated the non-standard application of ER-Tac (BID) with no side effects, no negative effects on glucose hemostasis monitored by fasting glucose, and no doubts about her adherence. Rather, her general condition improved against the background of having at least temporarily decelerated the rapid function decline of the kidney transplant. The transplant function is still stable 3 years after switching to ER-Tac twice daily (as of March 2024).

## 3 Discussion

To our knowledge, this is the first described case in the application of ER-Tac twice a day with beneficial effects in a kidney transplant recipient under extensive therapeutic drug monitoring.

The use of prolonged- or extended-release formulations of tacrolimus, usually taken once a day, is a part of the clinical standard of care for kidney transplant recipients (1). Recently, a meta-analysis indicated that the conversion from IR-Tac twice daily to ER-Tac once daily may decrease serum creatinine in kidney transplant recipients with a follow-up duration of more than 48 weeks, but at the same time, eGFR remained unchanged (11). At least many randomized controlled studies could show that the administration of ER-Tac once daily with less peak levels (and therefore in total reduced maximum plasma concentrations) does not appear to have an impact on either the efficacy or safety of this formulation and is an effective immunosuppressant treatment in kidney transplant recipients (7, 8, 12, 13). However, the nephrotoxicity of tacrolimus according to its peak levels and frequently unfavorable AUC with a risk of developing transplant glomerulopathy remains a limiting factor for graft survival, particularly in fast metabolizers (14, 15). This can be observed in immediate and extended-release formulations of tacrolimus (5, 6, 15).

Concerning therapeutic drug monitoring, the AUC is considered a better surrogate marker of systemic tacrolimus exposure than the maximum plasma concentration and is strongly associated with clinical outcomes (7). Furthermore, it is essential to identify the high peak levels of tacrolimus, which could cause renal toxicity. However, the determination of the AUC (including maximum concentration) for oral tacrolimus remains challenging and time-consuming in the clinical routine. Therefore, we performed only one AUC under each formulation for ER-Tac. Approaches of finger prick measurements might be a future option to easily include AUC in routine follow-up management of kidney transplant recipients (16). Other considerations in therapeutic drug monitoring include measuring tacrolimus concentrations in intracellular rather than whole blood. This is because most of the tacrolimus measured in whole blood is bound to erythrocytes and plasma proteins and thus represents the pharmacologically inactive fraction (17).

However, the off-label use of ER-Tac twice a day suggests critical discussion. *First,* in this case, after conversion to ER-Tac twice daily, trough levels increased (with two peaks), but the 24 h total AUC decreased (as seen in Figure 1A). This could be related to the improved transplant function with better eGFR. These potential beneficial effects must be weighed against the risk of long-term under immunosuppression and our patient’s history with histologically detected transplant glomerulopathy and preexisting DSAs (18). According to general data, chronic rejection is the major cause of death-censored transplant failure after kidney transplantation in the long-term follow-up (19). *Second,* several investigations revealed significantly higher peak levels and AUC of IR-Tac after the morning dose compared to after the evening dose, suggesting a circadian dependence in the clinical pharmacokinetics of tacrolimus (20). In the present case, the peak level in the evening was higher during the administration of ER-Tac twice daily, which could be due to the retard formulation. However, this requires further observation. *Third,* the application of ER-Tac once daily may improve patient adherence, which is an independent risk factor for the development of *de novo* DSAs (21). This advantage could be lost when taking ER-Tac twice a day. Therefore, the possibility of switching to the more frequent ER-Tac twice a day should be weighed carefully against possible patient’s non-adherence. Fourth, the absolute effect of the intervention seems quite modest. The reduction in AUC was in line with the dose reduction, although the switch to ER-Tac twice daily was accompanied by higher trough levels of tacrolimus. The improvement in renal function is probably due to the lower AUC, but other factors such as adherence, favorable hydration state or daily blood pressure, physical activity of the patient, or hyperfiltration of the kidney transplant might be involved. *Finally,* based on our observation, we hypothesize a particular advantage for high metabolizers taking ER-Tac twice daily. Thus, a further limitation of our case report is the absence of data on our patient’s specific CYP genotyping. However, the C/D ratio for IR-and ER-Tac strongly suggests fast tacrolimus metabolism in our patient (6). Our case report should focus on transplant recipients requiring high doses of extended- or prolonged-release tacrolimus, which are likely to result in high peak levels when taken as a single dose. Distributing the administration into two doses can result in lower peak and higher trough levels, thereby avoiding unnecessary dose increase and toxicity.

In conclusion, the administration of ER-Tac twice daily (BID) might be beneficial for selected patients with fast tacrolimus metabolism. However, in this special application, careful therapeutic drug monitoring including AUC is necessary and the transplant community should critically discuss this off-label drug use. As of today, the patient was converted 3 years ago and the kidney transplant function is stable, but the follow-up course of the transplant under ER-Tac twice a day needs to be further monitored closely.

## Data availability statement

The raw data supporting the conclusions of this article will be made available by the authors, without undue reservation.

## Ethics statement

Written informed consent was obtained from the individual(s) for the publication of any potentially identifiable images or data included in this article.

## Author contributions

LF: Formal analysis, Writing – original draft. LK: Data curation, Writing – review & editing. KN: Formal analysis, Methodology, Writing – review & editing. TS: Visualization, Writing – review & editing. MJS: Funding acquisition, Supervision, Writing – review & editing. DK: Supervision, Writing – review & editing, Funding acquisition. BM: Writing – review & editing, Supervision. MS: Writing – review & editing, Formal analysis, Methodology, Software. MF: Conceptualization, Supervision, Writing – review & editing. SK: Conceptualization, Data curation, Formal analysis, Investigation, Writing – original draft.

## Glossary

Ah: arteriolar hyalinosis
aah: hyaline arteriolar thickening
ABMR: antibody-mediated rejection
ATG: anti-thymocyte globulins
AUC: area under the concentration–time curve
BID: bis in die
BMI: body mass index
C: concentration
Cg: glomerular basement membrane double contours
ci: interstitial fibrosis
ct: tubular atrophy
cv: vascular fibrous intimal thickening
D: dose
DSA: donor-specific antibody
eGFR: estimated glomerular filtration rate
ER-Tac: extended-release tacrolimus
g: glomerulitis
g: gram
HLA: human leukocyte antigen
IR-Tac: immediate-release tacrolimus
i: interstitial inflammation
i-IFTA: inflammation in the area of IF/TA
MFI: mean fluorescence intensity
mg: milligram
mm: mesangial matrix expansion
mTOR: mammalian target of rapamycin
ptc: peritubular capillaritis
SID: semel in die
t: tubulitis
ti: total inflammation
v: intimal arteritis
